# A scoping review of guidelines and resources to promote evidence based prescribing for older people with sensory impairment

**DOI:** 10.1017/S1463423625100613

**Published:** 2025-11-25

**Authors:** Brenda Clark Morrison, Eugene Asante, Marilyn R. Lennon, Margaret C. Watson

**Affiliations:** 1 Strathclyde Institute of Pharmacy and Biomedical Sciences, University of Strathclyde, Glasgow, Scotland; 2 Department of Computer and Information Sciences, University of Strathclyde, Glasgow, Scotland; 3 Digital Health and Care, Department of Computer and Information Sciences, University of Strathclyde, Glasgow, Scotland; 4 Health Services Research and Pharmacy Practice, Strathclyde Institute of Pharmacy and Biomedical Sciences, University of Strathclyde, Glasgow, Scotland

**Keywords:** Aged, hearing loss, multimorbidity, pharmacists, polypharmacy, vision disorders

## Abstract

**Aim:**

This review explored whether and how prescribers modify their prescribing behaviour for older people (≥65) with hearing, visual or dual impairment (hereafter referred to as sensory impairment) in primary care settings and identified what evidence sources exist to inform prescribing for these specific patient populations.

**Background:**

Older people with sensory impairment may experience substantial challenges with medicines management compared with older people without sensory impairment. The prevalence of sensory impairment and medicine use increases with age, as such, practitioners may need to consider how to modify their prescribing behaviour to improve the safe and effective use of medicines.

**Methods:**

This study was conducted to reflect the Joanna Briggs Institute [JBI] methodology for scoping reviews. Electronic databases were searched: MEDLINE (Ovid), EMBASE (Ovid), Cochrane Library, Cumulative Index to Nursing and Allied Health Literature (CINAHL), Google, and Google Scholar. Qualitative and quantitative studies were included if published between January 2012 and April 2023. Grey literature sources, including Google and Google Scholar, were also searched. Studies were eligible for inclusion if they focussed on prescribing behaviour for older people with sensory impairment in primary care settings. Independent duplicate data extraction was undertaken of details about the participants, concept, context, study methods, outcomes, and key findings relevant to the review question.

**Findings:**

A total of 3,590 records were identified through database searching and 10 full text articles were retrieved. Grey literature identified a further 61 records. On examination, none of the articles fulfilled the inclusion criteria for this review.

**Conclusions:**

This review has highlighted a gap in the evidence regarding prescribing for these high risk patient populations. There may be a need for the development of resources, such as evidence based guidelines, to support the safe and effective use of medicines for these specific patient populations.

## Introduction

According to the World Health Organisation [WHO] (2022a, 2022b), hearing impairment will affect one in four people globally by 2030, and one in five people in the UK (Hearing Link, 2023). The UK population is expected to increase to 69.2 million by 2030 (Office for National Statistics [ONS], 2020), with one in five people being over 65 years old. An estimated 18 million people in the UK, approximately one in three adults, will be affected by deafness, hearing loss, or tinnitus, and 1.2 million adults will have hearing loss severe enough to prevent understanding most conversational speech (Royal National Institute for Deaf People [RNID], 2024). This age group is also more likely to experience visual impairment, which is projected to affect 2.7 million people in the UK by 2030, rising to 4 million by 2050 (ONS, 2020). Additionally, nearly half a million people in the UK will have both sight and hearing loss (Venus-Balgobin, 2023). Of these, 418,000 will be over the age of 70 (Care England, 2022). These demographic trends may lead to an increase in the prevalence of sensory impairment in the UK and worldwide.

For the purposes of this review, sensory impairment is used to describe visual and or hearing impairment or loss, whether present from birth or occurring later in life, ranging from a low level of loss to a more profound impairment. The term hard-of-hearing describes individuals with partial hearing loss who may use hearing aids and rely on a combination of auditory and visual communication.

Multimorbidity, the presence of two or more long term health conditions, has been estimated to affect up to 95% of the primary care population aged 65 years and older (Barnett *et al.,* 2012; Navickas *et al.,* 2016; Yarnall *et al.,* 2017). Compared with those with no long term health conditions, multimorbidity can affect an older person’s ability to access and use health care services, communicate with health care providers, and manage their medications (Barnett *et al.,* 2012; Yarnall *et al*., 2017; Smith *et al.,* 2019; Cooper *et al.,* 2023). Despite the increasing numbers of older people with multimorbidity, clinical practice guidelines and delivery of care are still built around single diseases, which can increase the risk of experiencing adverse drug events (Navickas *et al.,* 2016; Yarnall *et al*., 2017). This highlights the need for evidence based guidelines that deliver a person centred approach, better care management, enhanced multidisciplinary teamwork, and support for patient education (Navickas *et al.,* 2016). Such guidelines should consider the complexity and diversity of individual health needs, rather than focusing on individual diseases, made possible through the promotion of medicines optimization, deprescribing, and regular medication reviews (Barnett *et al.,* 2012; Picton and Wright 2013; Navickas *et al.,* 2016; Yarnall *et al.,* 2017; Tarrant *et al.,* 2023).

The prevalence of multimorbidity increases substantially with age (Navickas *et al.,* 2016). Older people with multimorbidity and sensory impairment may struggle to manage the multiple medications prescribed to them, leading to difficulties with treatment adherence (Alhusein *et al.,* 2018). Potentially inappropriate prescribing can introduce the risk of medication related adverse events, hospital admissions, falls, depression, and mortality (Riordan 2017; Yarnall *et al.,* 2017; Alhusein *et al.,* 2019). Evidence shows that older people with sensory impairment experience multiple challenges with all aspects of their medicine journey, from the consultation when a medicine is prescribed, to ordering, obtaining, storing medicine, and its administration and disposal (Smith *et al.,* 2019).

Older people with visual impairment face substantial challenges in their medicines management such as, identifying individual medicines, especially when their shape, size, or colour changes and/or have difficulty reading medication labels and information sheets (Alhusein *et al.,* 2019; Cooper *et al*., 2023). Older people may be unable to use assistive devices such as multi compartment devices, for example, dosette boxes, which are unsuitable for complex medication regimens, or live alone with no support, which may increase the risk of inappropriate medicines use, such as missed doses or unintentional overdose (Alhusein *et al.,* 2019; Cooper *et al*., 2023). These challenges might reduce the benefits and increase harms from medicines. Many types of health professionals are permitted to prescribe, including doctors and non-medical prescribers. The latter have been introduced in many countries to meet the increasing demand and need for healthcare (Edwards *et al*., 2022). The extent to which prescribers are trained and/or modify their prescribing behaviour for older people with sensory impairment is unknown.

## Aim

The aim of this review was to explore whether and how practitioners modify their prescribing behaviour for older people (≥ 65 years) with sensory impairment in primary care settings and the evidence sources available to inform prescribing for these specific patient populations.

The specific objectives were to:

1. Explore whether and how independent prescribers modify their prescribing behaviours for older people (65 years and over) with sensory impairment in primary care;

2. Identify potential factors that may influence prescribing behaviours, including barriers and facilitators to modifying prescribing for older people with sensory impairment;

3. Identify guidelines that may support evidence based prescribing for the safe and effective use of medicines by these specific patient populations; and

4. Identify information and resources that could influence prescribing for older people with sensory impairment.

## Methods

The review was conducted to reflect the Joanna Briggs Institute [JBI] (Peters *et al.,* 2020) methodology for scoping reviews and is reported according to the Preferred Reporting Items for Systematic reviews and Meta-Analysis extension for scoping reviews (PRISMA ScR) (Tricco *et al*., 2018; Page *et al*., 2021). The review protocol was registered with the Open Science Framework [OSF], registration number: osf.io/zy5we (Morrison *et al*., 2023).

### Eligibility criteria

The population, concept, context (PCC) framework was used to define the inclusion/exclusion criteria (Table 1) (Munn *et al*., 2018).

**Table 1.** Inclusion and exclusion criteria (Munn *et al*., 2018)

Population	• Older people (≥65) with sensory impairment including hearing, visual or dual impairment, living in the community, and who use medicines. • Registered independent prescribers’ working in primary care who provide care for older people (≥65) with sensory impairment. An independent prescriber can prescribe on their own initiative any medicine within their scope of practice and relevant legislation. • Studies that focus on individuals who reside in residential or nursing care homes will be excluded, as will studies involving children only and individuals aged 16–64 years. Additionally, studies published prior to 2012 will be excluded, as this date reflects key developments in prescribing policy and practice relevant to older adults with sensory impairment (UK Government, 2012; National Prescribing Centre & NICE, 2012; Shaikh, 2024).
Concept	• Exploration of how independent prescribers modify prescribing for older people (≥65) with sensory impairment. • Information and/or resources that informed prescribing for older people with sensory impairment. • Studies that do not explore prescribing practices, decision-making, or resources specific to older adults with sensory impairment will be excluded.
Context	• Prescribers in primary healthcare settings including General Practitioners (GPs), Nurses, Pharmacists, Dentists, Opticians, Physiotherapists, and Podiatrists. • Studies conducted outside of primary care settings, such as secondary or tertiary care, will be excluded.

### Information sources

Sources searched included the following electronic databases: MEDLINE (Ovid), EMBASE (Ovid), Cochrane Library, Cumulative Index to Nursing and Allied Health Literature (CINAHL). Both qualitative and quantitative studies were sought. Editorials and opinion articles were excluded.

### Grey literature

A wide range of grey literature sources were searched (Appendix 1) including local, national, and international organisations’ websites, health related and scientific organisations, professional organisation websites, and other information sources. Google and Google Scholar were also searched.

### Search strategy

The search strategy comprised keywords and subject headings contained in the titles and abstracts and was developed in consultation with institutional subject Librarian. The MEDLINE search strategy is presented in Appendix 2. The strategy was adapted for each of the five electronic databases. All databases were searched for the period January 2012 to 14 April 2023. The review was not limited by language or geographical region. One study was published in German. Non-English language studies were translated using Google translate.

### Source of evidence selection

The search results were uploaded into Rayyan systematic review software (Ouzzani *et al*., 2016) and duplicates removed. Titles and abstracts were screened by one author Brenda Morrison (BM) with duplicate, independent screening undertaken on a 10% random sample by another individual Eugene Asante (EA). Studies deemed relevant were included in the full text review. Duplicate, independent screening of the full text articles was undertaken (BM, EA). Disagreements were resolved by a third reviewer Margaret Watson (MW). The reference lists of all included studies were screened for additional studies.

## Results

**Figure 1.**
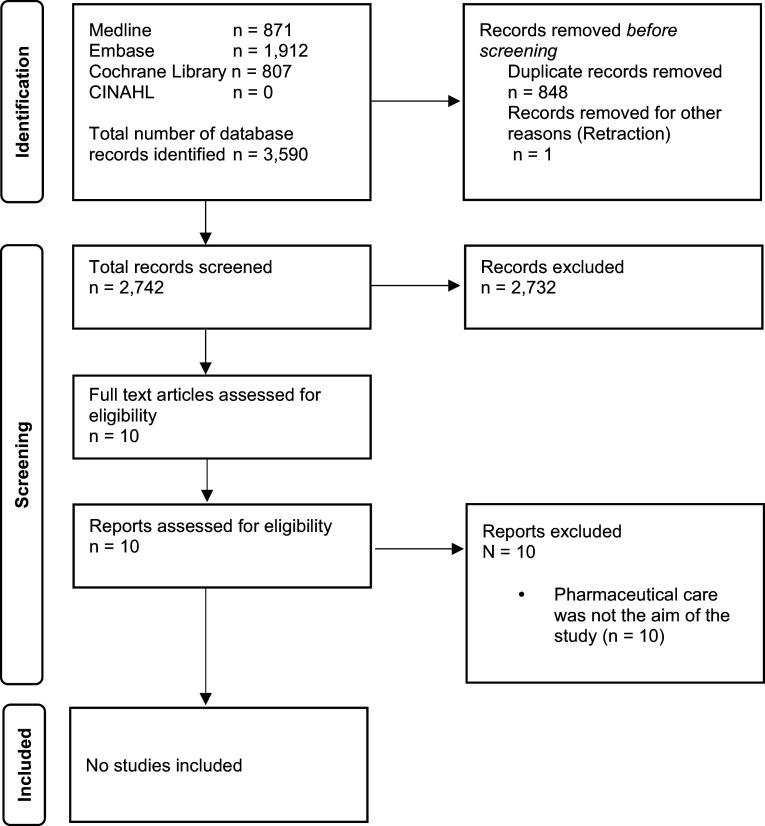
The preferred reporting items for systematic reviews and meta analysis extension for scoping reviews (PRISMA ScR) flow diagram (Tricco *et al*., 2018; Page *et al*., 2021).

A total of 3,590 records were identified through database searching and 10 full text articles were retrieved after screening. Grey literature identified a further 61 records. On examination of all full text articles, none fulfilled the inclusion criteria for this review (Figure 1). Hand searching of reference lists did not identify additional relevant studies.

Although no studies met the inclusion criteria, the literature highlights growing interest in ageing, sensory impairment, and healthcare delivery, with studies addressing communication strategies (McKee *et al.,* 2015; Stevens *et al.,* 2019), medication adherence (Smith & Bailey, 2014; Waterman *et al.,* 2013), access barriers (Lesch *et al.,* 2019), and sensory environments (Drahota *et al.,* 2012). Some emphasize the need for tailored treatment and integrated care for older adults with sensory impairment (Dietlein *et al.,* 2016; Jaiswal *et al.,* 2023). However, many focus on broader populations or hospital-based settings, which fall outside the scope of this review. To maintain relevance to prescribing in primary care for older adults (≥65 years), studies not addressing prescribing practices, decision-making, or relevant resources were excluded. Transferable insights, particularly around communication and adherence, may still inform future research and practice.

## Discussion

This review did not identify any studies or resources that met the inclusion criteria, which highlighted a notable gap, despite the prevalence of age related sensory impairment.

Several resources were identified that are relevant to more generic aspects of prescribing and for older people, in particular. For example, in the UK, the Royal Pharmaceutical Society has practice guidance for medicines optimization (Picton and Wright, 2013), defined as a person centred approach to safe and effective medicines use. Medicines optimization builds upon four principles: understanding the patient experience; evidence based choice of medicines; ensuring medicines use is as safe as possible; and making medicines optimization part of routine practice (Picton and Wright, 2013). Prescribers may be able to support older people with sensory impairment by following these principles and adopting strategies to support their medicines use, to ensure that people get the right choice of medicines, at the right time, and are engaged in the process by their healthcare professional.

Additional tools and guidelines were identified including the Medicines for Older People, part of the National Service Framework for Older People (England) (Department of Health [DoH], England, 2001), and the STOPP/START criteria (O’Mahony *et al*., 2014). The STOPP/START criteria is a set of guidelines to help identify and avoid potentially inappropriate prescribing and potential prescribing omissions in older people. The Beers Criteria (Samuel, 2023) is a list of guidelines published by the American Geriatrics Society [AGS] to help improve the safety of prescribing medications for older people. It identifies medications that should be used with caution in older people, especially those with certain health conditions. All the above tools aim to help avoid inappropriate prescribing and reduce the risk of polypharmacy and adverse drug events in older people.

Healthcare professionals who work with older people with sensory impairment, and who prescribe medicines, may need more training and education on how to help these specific patient populations to use their medicines safely and effectively. The lack of resources identified by this review could indicate a general lack of awareness of the needs of older people with sensory impairment in relation to their medicines. A free, online course was developed by some of the authors of this review to raise awareness of the challenges experienced by older people with their medicines, however, it does not focus specifically on prescribing (Future Learn, 2022).

Older people with sensory impairment, particularly those with hearing loss, might also benefit from longer healthcare consultations to address communication barriers (Stevens *et al.,* 2019), which often receive less attention and/or fewer adaptations compared with those for the visually impaired in healthcare settings (Reed *et al.,* 2020). Evidence suggests that the perspectives and experiences of deaf people, who have a distinct culture, language, and communication needs, should be recognised, and accommodated in relation to medicines use and prescribing practices (McKee *et al*., 2015; Lesch *et al*., 2018; Stevens *et al*., 2019).

Longer consultations might enable prescribers to explore supportive ways to tailor medication regimen for their older patients with sensory impairment. For example, they could explore patient preference for type of formulations and route of administration and involve the patient and their carers in shared decision-making. Simplified regimens can improve adherence and are beneficial for individuals with additional impaired motor and/or cognitive function (Smith and Bailey, 2014; Dietlein 2015). There is empirical evidence that the use of fixed combination preparations can simplify dosing regimens with no loss of therapeutic effect (Waterman 2013; Smith and Bailey, 2014; Dietlein 2015). Whilst there is limited empirical evidence of the effect of technologies and devices to support medication management by older people with sensory impairment, this is a vast range of low to high technology products and strategies that could be used to support their medication management (Cooper *et al*., 2023). Prescribers may not be fully aware of the range of devices and options available to help support their patients.

### Strengths and limitations

Standard scoping review methods were used and the study is reported in compliance with the PRISMA ScR checklist. A broad range of databases and other sources were searched. Searches were not limited by geography or language, yet despite the inclusive nature of the review, no studies or resources fulfilled the selection criteria. Whilst the review returned no included studies or resources, this is unlikely to be due to a limited search strategy or protocol, but may reflect a lack of available data on this topic.

The narrow inclusion criteria, focused specifically on prescribing in primary care for older people with sensory impairment, may have contributed to the absence of eligible studies. This highlights a notable evidence gap in the literature. While related studies offer insights into communication, adherence, and care environments, they often target broader populations or settings outside the review’s scope. Despite the lack of eligible studies, the review highlights the need for focused research in this area. Future studies may benefit from using broader inclusion criteria or alternative methods to gather relevant evidence.

## Conclusions

There is a paucity of guidance or other resources to support and inform prescribing for older people with sensory impairment. This review highlights a potential need for the development of resources, for example, evidence based guidelines, to support the safe and effective use of medicines for these specific patient populations. There may be a need for greater understanding of prescribers’ capacity, opportunity, and motivation to modify their prescribing, and their willingness to use prescribing resources to optimize medicines use and improve health outcomes for older people with sensory impairment.

## Appendix 1 Resources searched to identify potential studies from January 2012 to April 2023

**Table:**

Electronic Databases	MEDLINE (Ovid)
	EMBASE (Ovid)
	Cochrane Library
	Cumulative Index to Nursing and Allied Health Literature (CINAHL)
Web Search Engines	Google
	Google Scholar
Grey Literature	BASE (Bielefeld Academic Search Engine)
Resources	MedNar
OpenGrey	
	Web of Conferences
Professional	British Geriatrics Society
Organisations	British Dental Journal
	British Medical Association
	College of Optometrists
	General Dental Council
	General Optical Council
	General Pharmaceutical Council
	Health and Care Professions Council
	The Nursing and Midwifery Council
	Royal College of General Practitioners
	Royal College of Nursing
	Royal Pharmaceutical Society
	Specialist Pharmacy Service
Scientific	BioMed Central
Organisations	Elsevier Science Direct
	The Oxford Review
Health-related	British Medical Journal
Organisations	British National Formulary
	Care Quality Commission
	Department of Health, England
	East Lancashire Hospital NHS Trust
	Greater Glasgow and Clyde National Health Service Trust
	Guidelines International Network
	Health Improvement Scotland
	Healthcare Management Information Consortium
	National Centre for Complementary and Integrative Health
	Scottish Intercollegiate Guidelines Network
	The Canadian Agency for Drugs and Technologies in Health
	The King’s Fund Information and Library Service
	The National Institute for Care and Health Excellence
	The National Technical Information Service
	World Health Organisation
Other Resources	Age UK
	American Foundation Fighting Blindness
	American Foundation for the Blind
	British Deaf Association
	Canadian National Institute for the Blind
	Community Northern Ireland
	DeafBlind UK
	Diabetes UK
	Disability Information Scotland
	European DeafBlind Network
	Hearing Link
	Hearing Loss Association of America
	Hi-Vis UK
	International Disability Alliance
	Macular Society
	National Federation for the Blind
	Older People’s Advocacy Alliance UK
	Sense
	Signature (formerly Council for the Advancement of Communication with Deaf People)
	SignHealth
	The Royal National Institute for the Blind
	The Royal National Institute for the Deaf
	Thomas Plocklington Trust UK

## Appendix 2 Search Strategy

**Data base:** Ovid MEDLINE(R) ALL <January 1, 2012 to April 14, 2023>

**Table:**

No.	Query
1.	Prescriber.ti,ab,kw.
2.	((Community or Nonmedical or Nonmedical Prescrib* or Nonmedical Healthcare) adj3 Pract*).ti,ab,kw.
3.	((Nurs* or Nurse Independent or Extended Formulary Nurse or Community Practitioner Nurse or Pharmacist or Pharmacist Independent) adj3 Prescrib*).ti,ab,kw.
4.	(Health* Professional or Health Care Professional or Healthcare Professional or Health* Team or Health Care Team or Healthcare Team or Physician or Clinician or Provider* or Doctor or Caregiver or Care Provider or GP or General Practitioner or Therapist or Practitioner).ti,ab,kw.
5.	exp Pharmacists/
6.	exp Community Pharmacy Services/
7.	(Prescri* Drug* or Prescr* Medic* or Prescr* Pharma*).ti,ab,kw.
8.	(Drug* Therap* or Therap* Drug*).ti,ab,kw.
9.	exp Inappropriate Prescribing/
10.	((Appropriate or Optim* or Inappropriat* or Suboptim* or Sub-optim* or Unnecessary or Incorrect* or In-correct* or Excessive or Multiple or Concurrent*) adj2 (Medicine* or Medication* or Prescription* or Drug*)).ti,ab,kw.
11.	((Over adj1 Prescript*) or (Overprescrib* or Overprescript*)).ti,ab,kw.
12.	((Under adj Prescript*) or (Underprescrib* or Underprescript*)).ti,ab,kw.
13.	Medication Appropriateness Index.ti,ab,kw.
14.	(Quality adj (Prescribing or Prescription* or Medication*)).ti,ab,kw.
15.	(Improv* adj (Prescrib* or Prescription* or Pharmaco*)).ti,ab,kw.
16.	Medication Therapy Management/
17.	(Medication* Management or Medication* Therapy Management or Medication* Strategy or Medication* Strategies or (Medication* adj2 Review*)).ti,ab,kw.
18.	Drug Regimen Review*.ti,ab,kw.
19.	Drug Utilization Review/
20.	(Drug adj Utili*ation adj2 (Review* or Evaluat*)).ti,ab,kw.
21.	Drug Related Problem*.ti,ab,kw.
22.	((Prescribing or Prescription*) adj2 Pattern*).ti,ab,kw.
23.	Prescriptions/
24.	Drug Prescriptions/
25.	Prescription Drugs/
26.	((Hearing or Auditory or Auditory Process*) adj3 (Dysfunction* or Decline* or Disorder* or Loss or Defect*)).ti,ab,kw.
27.	(((Unilateral or Bilateral or Binaural or Aural or Functional or Conductive or High-Frequency or Mixed Conductive Sensorineural or Congenital or Sudden Sensorineural or Age-Related) adj3 Hearing Loss*) or Loss* Hearing).ti,ab,kw.
28.	(Profoundly Deaf or Profoundly Deaf without Speech or Non-Vocal or Partial* Deaf*).ti,ab,kw.
29.	exp Deafness/
30.	exp Vision Disorders/
31.	((((((Visual* or Vision or Vis* or Sight) adj3 (Impair* or Impair* Person or Loss* or Field Loss* or Loss* or Dysfunction or Disorder or Defect*)) or (Transient or Hemianopsia or Functional or Defective or Subacute or Hemisensory)) adj3 (Vis* or Vis* Loss*)) or (Impair* or Low or Loss* or Abnormal or Blurred or Person)) adj3 (Vis* or Visual* or Visual Impair* or Vision or Sight)).ti,ab,kw.
32.	(Blind* or Partial* Sight* or Blindness or Acquired Blind* or Congenital* Blind* or Adventitious Blind*).ti,ab,kw.
33.	exp Deaf-Blind Disorders/
34.	exp Usher Syndromes/
35.	exp Wolfram Syndrome/
36.	((((Dual-Sensory or Dual Sensory or Multi-Sensory or Multi Sensory or Dual or DSI or Sensory Process* or Somatosensory) adj3 (Impair* or Disorder* or Decline* or Loss* or Defect*)) or (Congenital or Acquired)) adj3 (DeafBlind* or Deaf Blind* or Deaf-Blind*)).ti,ab,kw.
37.	Geriatrics/
38.	(Gerentol* or Ageing or Aging or Elder* or Geriatric* or Seniors or Old Age or Late* Life).ti,ab,kw.
39.	(Older adj (Person* or People or Adult* or Patient* or Outpatient*)).ti,ab,kw.
40.	1 or 2 or 3 or 4 or 5 or 6 or 7 or 8 or 9 or 10 or 11 or 12 or 13 or 14 or 15 or 16 or 17 or 18 or 19 or 20 or 21 or 22 or 23 or 24 or 25
41.	26 or 27 or 28 or 29 or 30 or 31 or 32 or 33 or 34 or 35 or 36
42.	37 or 38 or 39
43.	40 and 41 and 42
44.	limit 43 to yr=2012-current
